# Case report: Selpercatinib in the treatment of *RET* fusion-positive advanced lung adenocarcinoma: a challenging clinical case

**DOI:** 10.3389/fonc.2024.1500449

**Published:** 2025-01-15

**Authors:** Raffaella Pagliaro, Paola Maria Medusa, Fabiana Vitiello, Luigi Aronne, Susan F. M. Campbell, Fabio Perrotta, Andrea Bianco

**Affiliations:** ^1^ Department of Translational Medical Sciences University of Campania L. Vanvitelli, Naples, Italy; ^2^ Clinic of Respiratory Diseases “Vanvitelli”, A.O. dei Colli, Monaldi Hospital, Naples, Italy; ^3^ Department of Pneumology and Oncology, Monaldi Hospital A.O. Dei Colli, Naples, Italy

**Keywords:** lung adenocarcinoma, *RET* fusion, selpercatinib, targeted therapy, precision medicine

## Abstract

**Background:**

Rearranged during transfection (*RET*) fusions represent a distinct molecular subset of non-small cell lung cancer (NSCLC) with targeted therapeutic potential. Selpercatinib, a highly selective *RET* inhibitor, has demonstrated efficacy in various solid tumors harboring *RET* alterations. Here, we present a case highlighting the use and clinical outcomes of selpercatinib in a patient diagnosed with advanced lung adenocarcinoma harboring a *RET* fusion.

**Case presentation:**

A 59-year-old woman with a history of stage IV lung adenocarcinoma harboring a *KIF5B-RET* fusion presented with disease progression following first-line chemo-immunotherapy. Selpercatinib was initiated as a targeted therapy, leading to a notable radiographic response and clinical improvement. The patient experienced a significant reduction in tumor burden and reported improved symptom control, with no significant adverse effects during the 21-month follow-up period.

**Conclusions:**

This case highlights the efficacy and tolerability of selpercatinib in treating advanced lung adenocarcinoma with a *RET* fusion. The observed clinical response supports the early use of selpercatinib as a targeted therapy for *RET* fusion-positive NSCLC, including in patients with compromised general and respiratory conditions, especially in cases refractory to conventional treatments. Long-term follow-up studies are warranted to validate these findings and assess the durability of responses.

## Introduction

Lung cancer remains the primary cause of cancer-related deaths globally, despite significant progress in understanding the aetiology and biology of tumors, as well as the role of enhancing immunologic control and the introduction of advanced treatment modalities (1, 2). Non-small cell lung cancer (NSCLC) is the most common type of lung cancer, accounting for 84% of all lung cancer diagnoses (3, 4). Although chemotherapy remains a common treatment (3, 5), targetable driver gene mutations have revolutionized the therapeutic landscape for patients with advanced NSCLC. The rearranged during transfection (*RET*) proto-oncogene encodes a transmembrane receptor tyrosine kinase involved in normal embryonic development. *RET* gene fusions or rearrangements occur in 1%–2% of NSCLC cases (6), particularly in younger (≤ 60 years), nonsmoking patients with adenocarcinoma. These rearrangements may make the cancer more responsive to certain chemotherapy drugs, such as pemetrexed (7). Malignant pleural effusions (MPE) in lung adenocarcinoma frequently harbor RET rearrangements (8). The detection of RET alterations is recommended to identify NSCLC patients who may be eligible for *RET* inhibitors. Molecular testing techniques available to detect *RET* rearrangement include next-generation sequencing (NGS), reverse transcription polymerase chain reaction (RT-PCR), fluorescence *in situ* hybridization (FISH), and immunohistochemistry (9), and the analysis of circulating tumor DNA (ctDNA) (10). Selpercatinib is a novel tyrosine kinase inhibitor that acts as a selective blocker of the activity of the *RET* protein and its variants. By inhibiting *RET*, selpercatinib helps to disrupt the signaling pathways that promote cancer growth and survival in cells where *RET* alterations are present (11). We report a challenging case of a patient treated with selpercatinib in the second line after failure of treatment with chemoimmunotherapy.

## Case report

**Figure 1:**
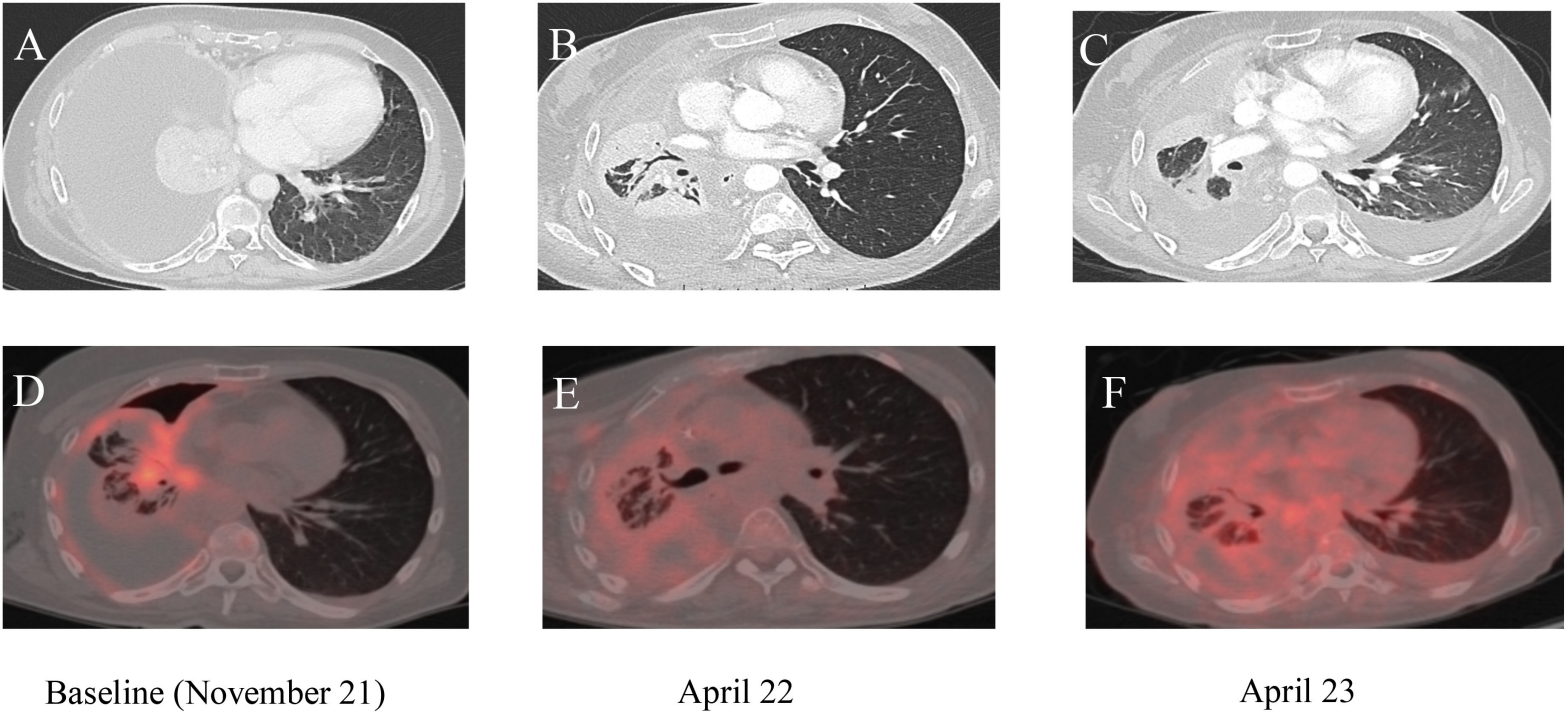
**(A–C)** Chest computed tomography (CT) images; **(D–F)** corresponding PET/CT scans. **(A, D)** Baseline imaging before the start of selpercatinib treatment. **(B, E)** Imaging results 2 months after the start of treatment. **(C, F)** Chest CT and PET/CT scans after 14 months of treatment with selpercatinib, highlighting changes in disease progression and response to therapy over time.

A 59-year-old female patient, a never-smoker, was admitted to our department for acute hypercapnic respiratory failure with right lung consolidation and pleural effusion. Her previous medical history included type II diabetes mellitus, treated with oral hypoglycemics. After thoracentesis, a whole-body CT scan at baseline exhibited a right lower lobe consolidation with enlarged mediastinal lymph nodes, right pleural effusion, and multiple spinal cord lesions. The patient was unable to move from a bed to a standing position, and blood gas analysis showed the presence of respiratory acidosis (pH 7.30), hypercapnia, hypoxemia, and raised lactate (1.8 mmol/L) at rest. Minimal exercise, including a change in position, resulted in severe worsening of hypoxemia. Following mechanical ventilatory support, a chest drain was inserted, and 2 L of brown-colored pleural fluid was removed, with a sample collected for cytological studies. Immunohistochemical analysis of the pleural fluid confirmed the diagnosis of adenocarcinoma and was positive for thyroid transcription factor 1, cytokeratin, low molecular weight cytokeratin, synaptophysin, plasma chromogranin A foci, and cytokeratin 20, and negative for p40 and natural killer CD56. The Ki-67 index was + 30%. Molecular profiling of a cellular sample of pleural effusion resulted in negative findings for epidermal growth factor (*EGFR*), anaplastic lymphoma kinase (*ALK*), and proto-oncogene tyrosine-protein kinase 1 (*ROS-1*); the programmed cell death-ligand 1 (*PD-L1*) tumor proportion score (TPS) on this sample was between 1% and 49%; *RET* fusion was not assessed on the tissue sample at this stage. The severe clinical condition of the patient meant that additional invasive procedures to complete diagnosis were contraindicated. A liquid biopsy using the FoundationOne^®^Liquid CDx assay was performed to analyze ctDNA and obtain genomic information through the NGS method. The panel included over 300 genes, such as *RET*, *EGFR*, *ROS-1*, *ALK*, mesenchymal-epithelial transition factor (*MET*), Kirsten rat sarcoma virus *(K-RAS)*, v-raf murine sarcoma viral oncogene homolog B (*BRAF*), tumor protein p53 (*TP53*), epidermal growth factor receptor 2 (*ERBB2*), and fibroblast growth factor receptor 2 (*FGFR2*). The results revealed *RET* fusion positivity in the patient’s blood sample. Therefore, the final diagnosis was lung adenocarcinoma, *RET* fusion-positive, and the clinical stage was IVB (T4N3M1c). Since the Eastern Cooperative Oncology Group performance status (ECOG-PS) at baseline was 4, the patient was only a candidate for best respiratory support care. The chest drain remained *in situ* for 21 days until the daily pleural drainage volume had sufficiently reduced. Regarding the acute respiratory failure, the patient was initially treated with oxygen therapy via nasal cannula, and due to the worsening of dyspnea, was quickly changed to a high-flow nasal canula (HFNC). The complexity of the case required alternating between HFNC and continuous positive airway pressure (CPAP) cycles. Despite this approach, the patient’s general condition continued to worsen, and blood gas analysis showed acute hypoxemic–hypercapnic respiratory failure with acidosis; therefore, noninvasive ventilation (NIV) with bi-level positive airway pressure (BiPAP) was required. After intensive cardiorespiratory support, a significant improvement in both general and respiratory clinical conditions was achieved (ECOG-PS 2), and the patient was able to start chemotherapy, including cisplatin and pemetrexed, as well as immune check point inhibitors (*ICIs*) (pembrolizumab). After two cycles of chemoimmunotherapy, the patient experienced severe hematological toxicity [thrombocytopenia and anemia grade 4 according to the Common Terminology Criteria for Adverse Events: CTCAE Ver.5 (12)] and a worsening of the general clinical condition; therefore, we decided to commence second-line therapy with selpercatinib, a tyrosine kinase inhibitor (TKI) selective for RET fusion, at 160 mg bid (ECOG-PS 0-1). However, the treatment plan was agreed upon with the patient. At restaging after 6 months of *RET* fusion TKI therapy, CT scans documented complete disease response (**Figure 1**), with no further accumulation of pleural fluid and no evidence of respiratory failure. During treatment with selpercatinib, the patient experienced grade 2 diarrhea and bilateral leg edema, which were managed with dietary changes, antidiarrheals, and diuretics, allowing selpercatinib therapy to continue.

**Figure 2:**
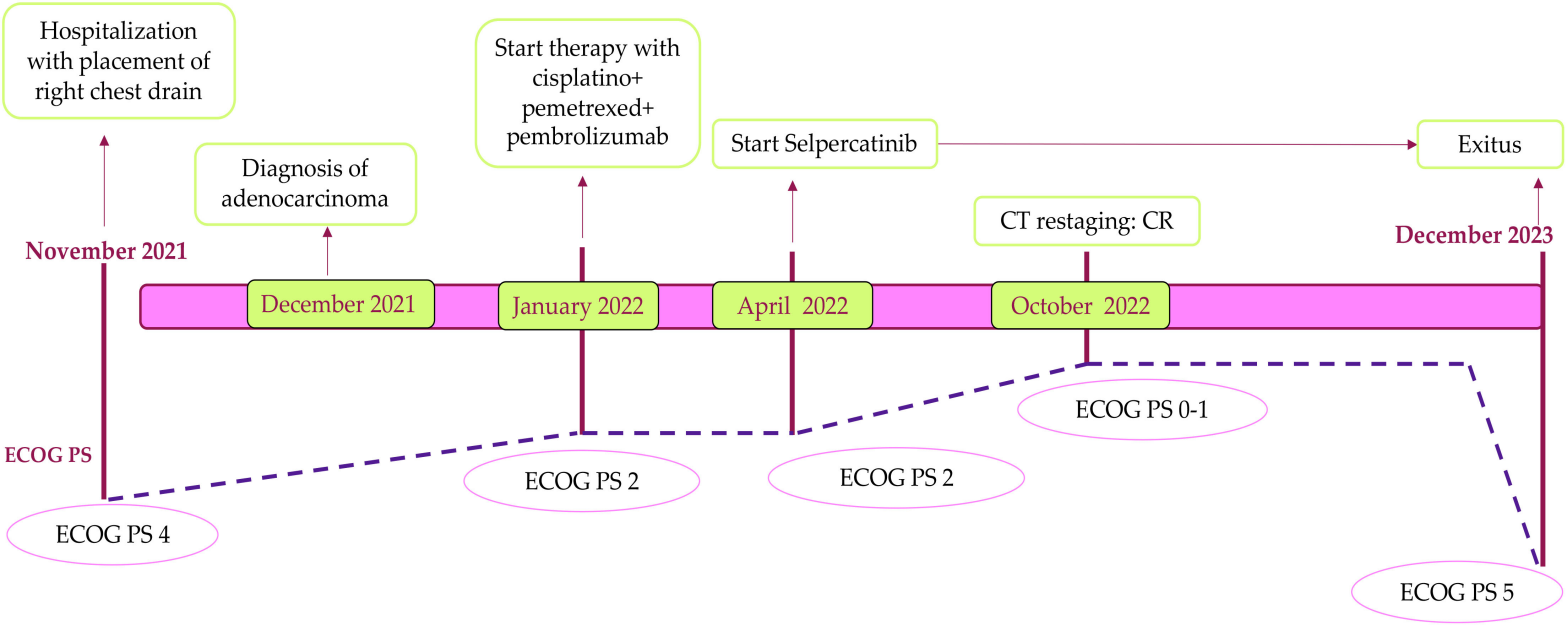
Patient timeline. This figure illustrates the chronological overview of the patient’s treatment and corresponding response, from the baseline in November 2021 through to the time of exit in December 2023. It highlights key points in the patient’s clinical course, including treatment interventions, response assessments, and significant changes in health status. CT, computed tomography; CR, complete response.

After 1 year of treatment with selpercatinib, liver function tests showed an increase in alanine aminotransferase (ALT) and aspartate aminotransferase (AST) (gastrointestinal (GI) toxicity grade 3 according to CTCAE ver.5 (12)), and the CT abdominal scans documented the presence of free fluid in the abdomen. The selpercatinib dose was reduced to 80 mg bid, with transaminase levels dropping to grade 1 within 2 weeks. Therefore, empiric antibiotic therapy was started, and a sample of ascitic fluid was collected for cytological analysis; it showed exclusively fibrin-blood material. After 1 week, the abdominal pain worsened, and abdominal drainage was performed with the removal of approximately 2 L of ascitic fluid. At restaging, the CT confirmed a complete response of the target lesion and an improvement of ascites; therefore, the selpercatinib dose was retitrated to 160 mg bid, and the patient continued selpercatinib medication for about 21 months without major toxicity. The patient died due to metastatic spread of disease to the omentum and peritoneal carcinomatosis, which led to worsening of liver failure. The timeline of treatment is shown in **Figure 2**.

## Discussion

This report presents the case of a severely respiratory-compromised patient with grade IV NSCLC harboring a *RET* fusion mutation, treated with second-line selpercatinib, resulting in a durable response. The patient initially presented with severe clinical conditions (ECOG-PS of 4 at baseline), requiring complex clinical management of respiratory failure and pleural effusion. Significant improvement in clinical status allowed for the initiation of active treatments. During treatment with selpercatinib, grade 3 elevations in transaminase levels and ascites were successfully managed by optimizing the drug dose.

Molecular assessment for *RET* alterations in NSCLC patients is highly recommended to identify those patients who could potentially benefit from strategies tackling *RET* inhibition. Various molecular testing methods are available for detecting *RET* rearrangements. Additionally, the analysis of ctDNA can detect *RET* rearrangements, offering several benefits such as the ability to test for multiple molecular alterations at once and avoid invasive procedures for patients. Liquid biopsy can be applied to all stages of cancer diagnosis and treatment, allowing noninvasive and real-time monitoring of disease development. To detect these, the NGS method can identify both known and unknown variants exploring genome-wide DNA variations, detecting mutations with a minor allele frequency (MAF) as low as < 1% (13, 14). NGS is increasingly used with targeted panels for highly sensitive detection of specific ctDNA mutations whereas whole genome sequencing (WGS) provides a comprehensive tumor genomic profile, but it is costly and limited when ctDNA concentrations in blood samples are low (15). By enhancing biomarker testing and utilizing targeted therapies, precision medicine can be advanced, although access to these resources may differ between countries. In 2020, the second-generation *RET*-specific TKIs selpercatinib and pralsetinib were approved by the FDA for *RET* fusion NSCLC based on the LIBRETTO-001 and ARROW clinical trials (16, 17). In particular, selpercatinib is a first-in-class, highly selective, and potent *RET* kinase inhibitor with CNS penetration. An updated assessment of the efficacy and safety of selpercatinib in patients with *RET* fusion-positive NSCLC treated in the phase I/II LI-BRETTO-001 trial showed an overall response rate (ORR) of 84% and 61% in treatment-naive patients and patients with prior platinum-based chemotherapy, respectively. The median progression-free survival (PFS) was 22.0 months for treatment-naive patients and 24.9 months for platinum doublet-pretreated patients (18). Similarly, pralsetinib is a selective and highly potent small molecule inhibitor of wild-type *RET* and mutated or rearranged *RET*, with activity against V804 gatekeeper mutations that confer resistance to multikinase inhibitors (19). Regarding safety, the most common high-grade treatment-related adverse events (TRAE) for selpercatinib were high blood pressure (14%), elevated levels of alanine transaminase (ALT) (13%), and aspartate aminotransferase (AST) (10%), while neutropenia (18%), high blood pressure (11%), and anemia (10%) were common for pralsetinib. Most of these side effects did not require drug interruption; however, 30% (selpercatinib) and 38% (pralsetinib) necessitated dose reduction, while a small percentage had to discontinue treatment. Rarely, chylous ascites (CA) may occur as a side effect of *RET* TKI treatment (20). Given the high efficacy of selective *RET* inhibitors, it is important to manage side effects to prevent treatment interruption and compromising results. In particular, TKI dose adjustments did not significantly reduce the reaccumulation of effusions (21); however, the interpretation of this result is limited by the small sample size and factors such as home care of indwelling catheters, according to the literature review (22). It will be crucial to assess the effectiveness of different management approaches for chylous effusions in a larger group of patients. In general, for grade ≥ 3 hepatotoxicity, the RET inhibitor should be paused with regular monitoring of ALT/AST until improvement to grade 1, at which point it can be resumed at a reduced dose. In the case of recurrence of hepatoxicity grade ≥ 3, selpercatinib must be discontinued (23). In summary, adverse events of TKIs are usually dose-dependent; the toxicity and effectiveness of TKIs are often linked, meaning that the toxic side effects caused by these drugs can serve as indicators of successful pharmacological inhibition (24). The combined effects of both on- and off-target toxicities can reduce the patient’s quality of life and potentially limit the effective dose of medication (25). Factors such as drug interactions, genetic variations, patient adherence, and the drug’s absorption, distribution, metabolism, and elimination processes must be considered to determine the optimal dosage for each patient (26). Identifying biomarkers that can predict or monitor these toxicities is crucial for optimizing treatment and improving patient outcomes. Some potential biomarkers of toxicity during TKI therapy include circulating cytokines, such as IL-6 and TNF-alpha, which have been associated with inflammatory side effects like skin rashes, gastrointestinal disturbances, and fatigue (27, 28). Monitoring these biomarkers can help identify patients at higher risk for adverse effects, allowing for early intervention and more tailored therapy.

Selpercatinib and pralsetinib are preferred first-line therapy options for patients with *RET* fusion-positive metastatic NSCLC and are recommended as subsequent therapies if RET inhibitors have not been used in the first-line setting (29). The phase III LIBRETTO-431 trial was designed to define the optimal first-line regimen for patients with *RET* fusion-positive NSCLC.

## Conclusions

In conclusion, this case report emphasizes the importance of genomic testing across multiple matrices, including liquid biopsy, before excluding any driver gene mutations. It highlights the efficacy and safety of selpercatinib for *RET* fusion-positive NSCLC. The observed clinical response of our patient further supports the effectiveness of selpercatinib in treating *RET* fusion-positive NSCLC, even in patients with severe clinical conditions. The long-term durability of response and survival in this challenging case highlights the importance of initiating targeted therapy early for *RET* fusion-positive patients.

## Data Availability

The original contributions presented in the study are included in the article/supplementary material. Further inquiries can be directed to the corresponding authors.
